# Supplementary figures and images for: IL-18 binding protein can be a prognostic biomarker for idiopathic pulmonary fibrosis
Source: PLoS One. 2021 Jun 4;16(6):e0252594. doi: 10.1371/journal.pone.0252594 (PMC8177514; doi:10.1371/journal.pone.0252594)

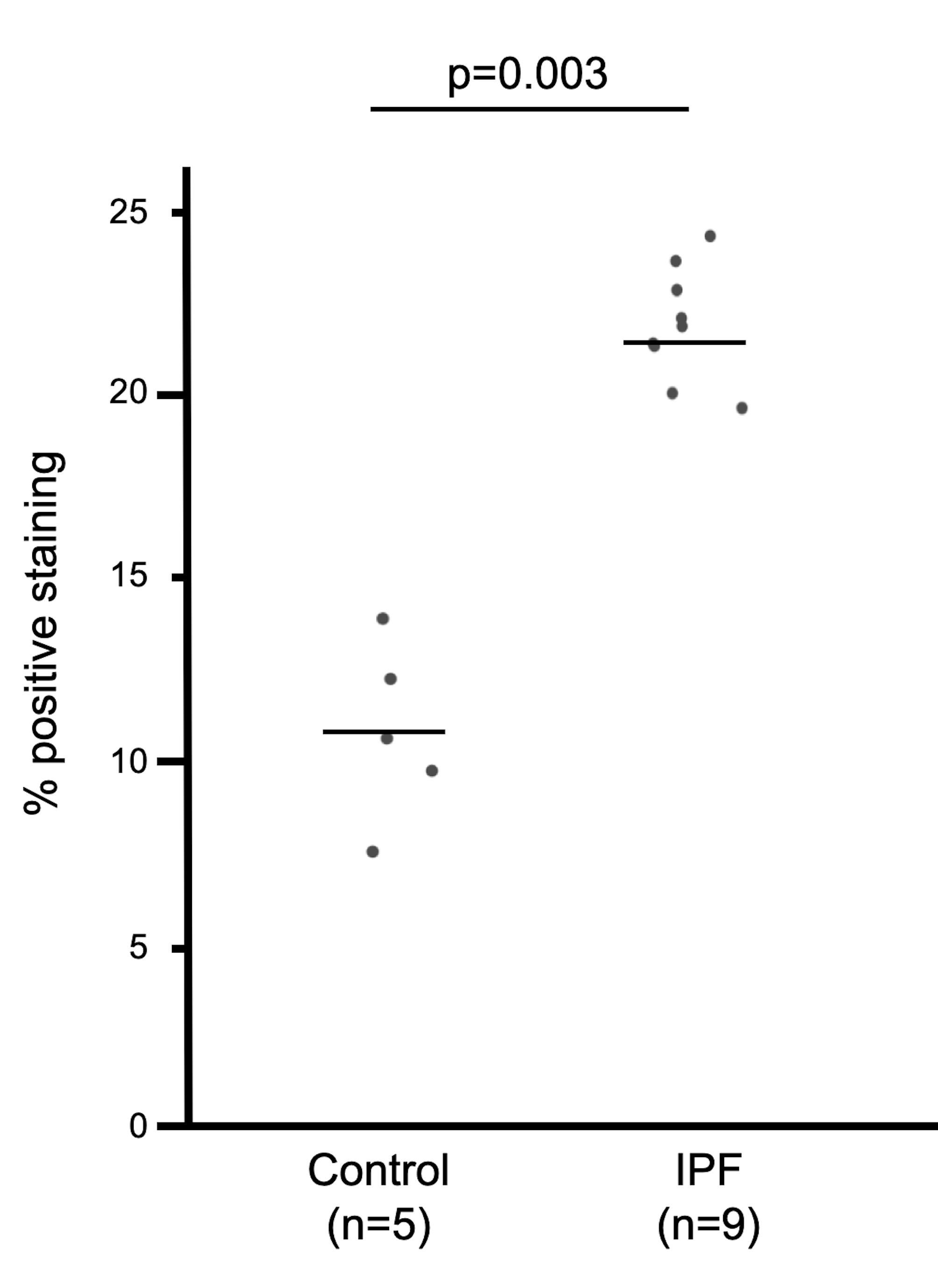

Supplement: S1 Fig — The percentage of the positively stained area was higher in patients with IPF (21.7% [IQR: 20.6–23.1]) than those in control lung (10.6% [IQR: 7.5–13.0]) (p = 0.003). Horizontal bars represent median. IL-18BP: interleukin-18 binding protein. (TIF) [file pone.0252594.s001.tif]

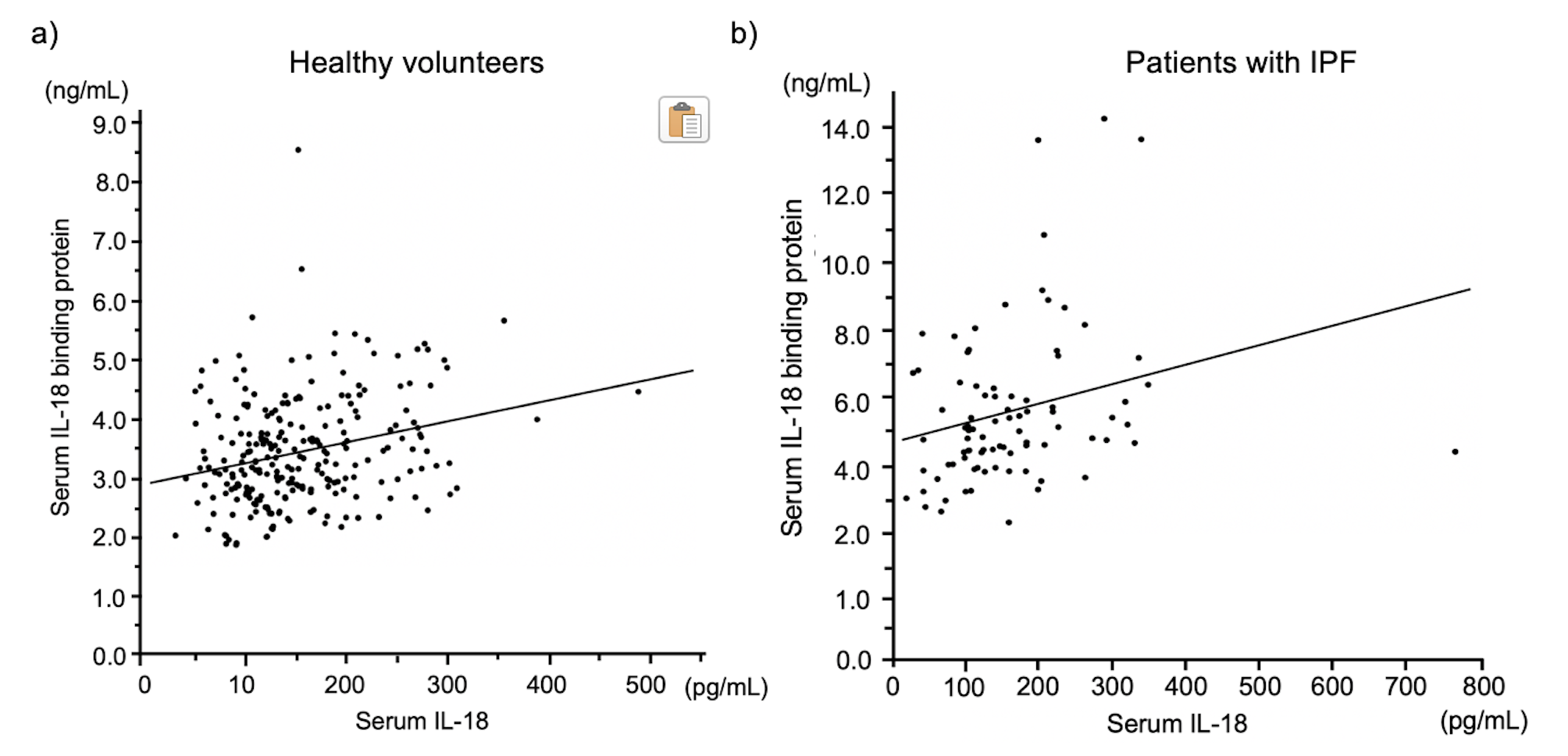

Supplement: S2 Fig — (a) Serum IL-18BP and IL-18 levels showed significant positive correlations in HVs (Spearman r = 0.267, p < 0.001). (b) Serum IL-18BP and IL-18 levels showed significant positive correlations in IPF patients (r = 0.251, p = 0.019). IL-18BP: interleukin-18 binding protein, IL-18: interleukin-18, HVs: healthy volunteers, IPF: idiopathic pulmonary fibrosis. (TIF) [file pone.0252594.s002.tif]

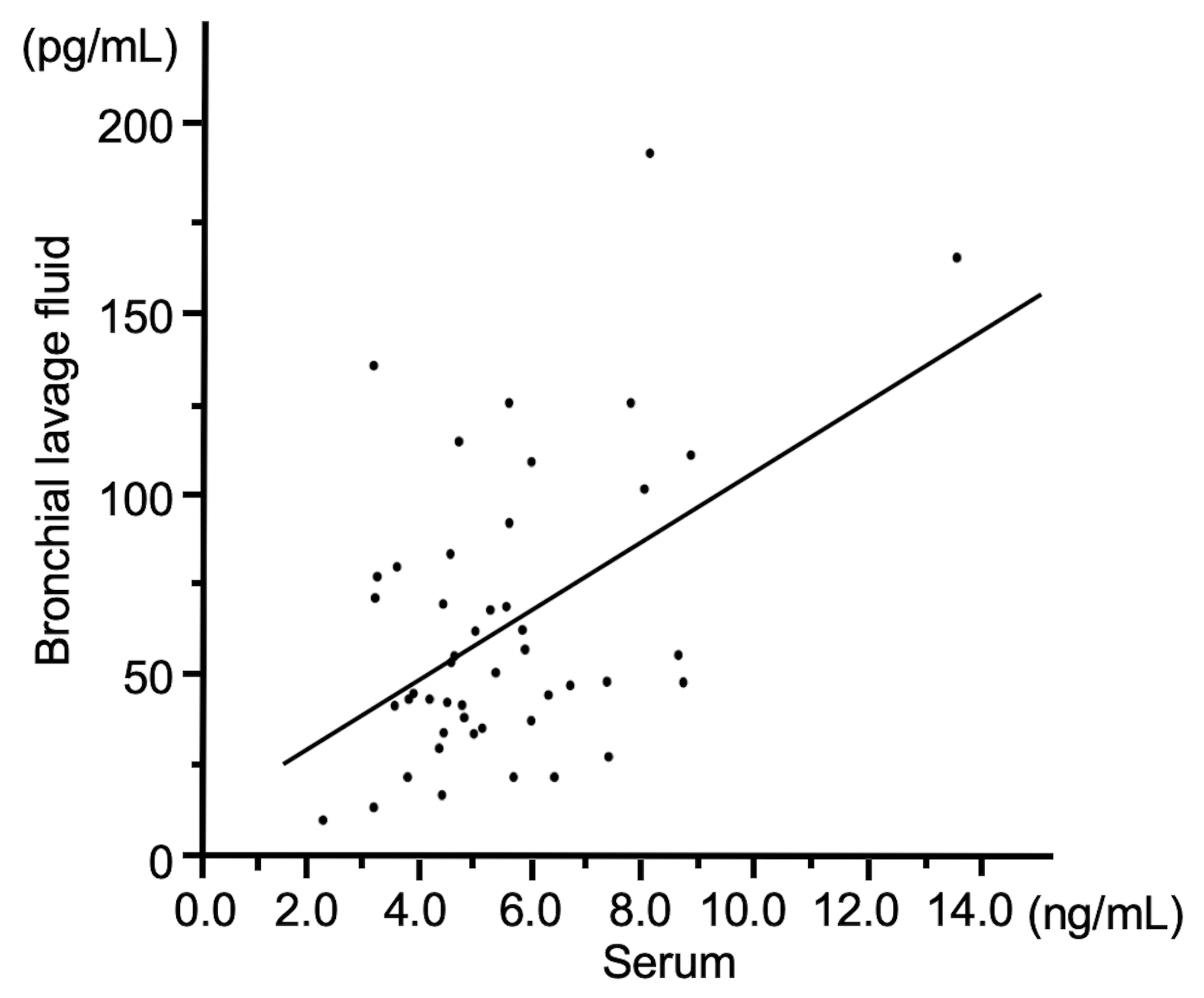

Supplement: S3 Fig — Serum IL-18BP and BALF IL-18BP levels showed significant positive correlations in IPF patients (Spearman r = 0.406, p = 0.005). IL-18BP: interleukin-18 binding protein, BALF: bronchoalveolar lavage fluid, IPF: idiopathic pulmonary fibrosis. (TIF) [file pone.0252594.s003.tif]

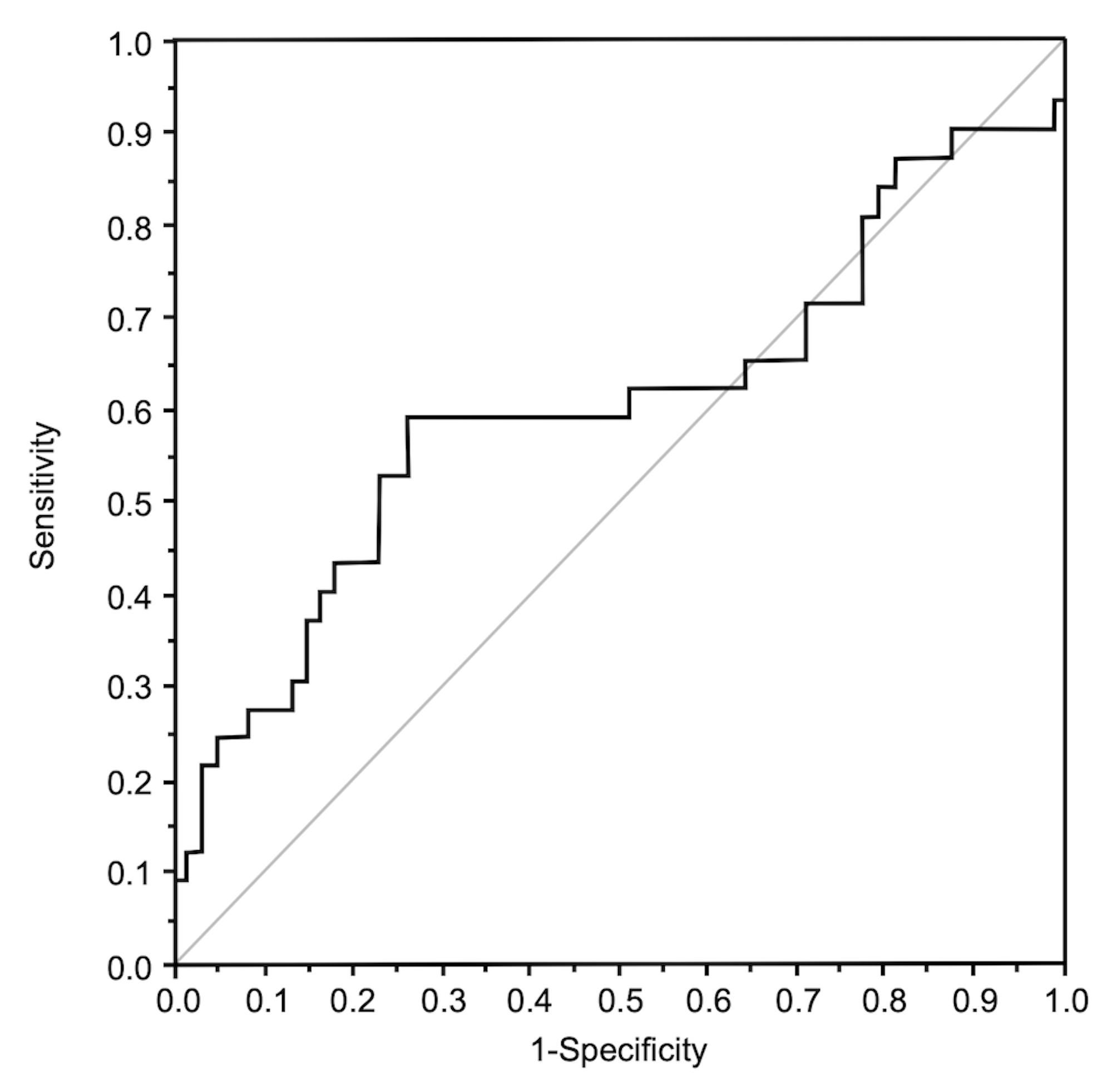

Supplement: S4 Fig — ROC analysis of serum IL-18BP levels was performed between those that have died and those that have survived or censored during three years from diagnosis (AUC 0.610, 95% Cl: 0.429–0.732). ROC: receiver operating characteristic, IL-18BP: interleukin-18 binding protein, IPF: idiopathic pulmonary fibrosis, AUC: area under the curve. (TIF) [file pone.0252594.s004.tif]

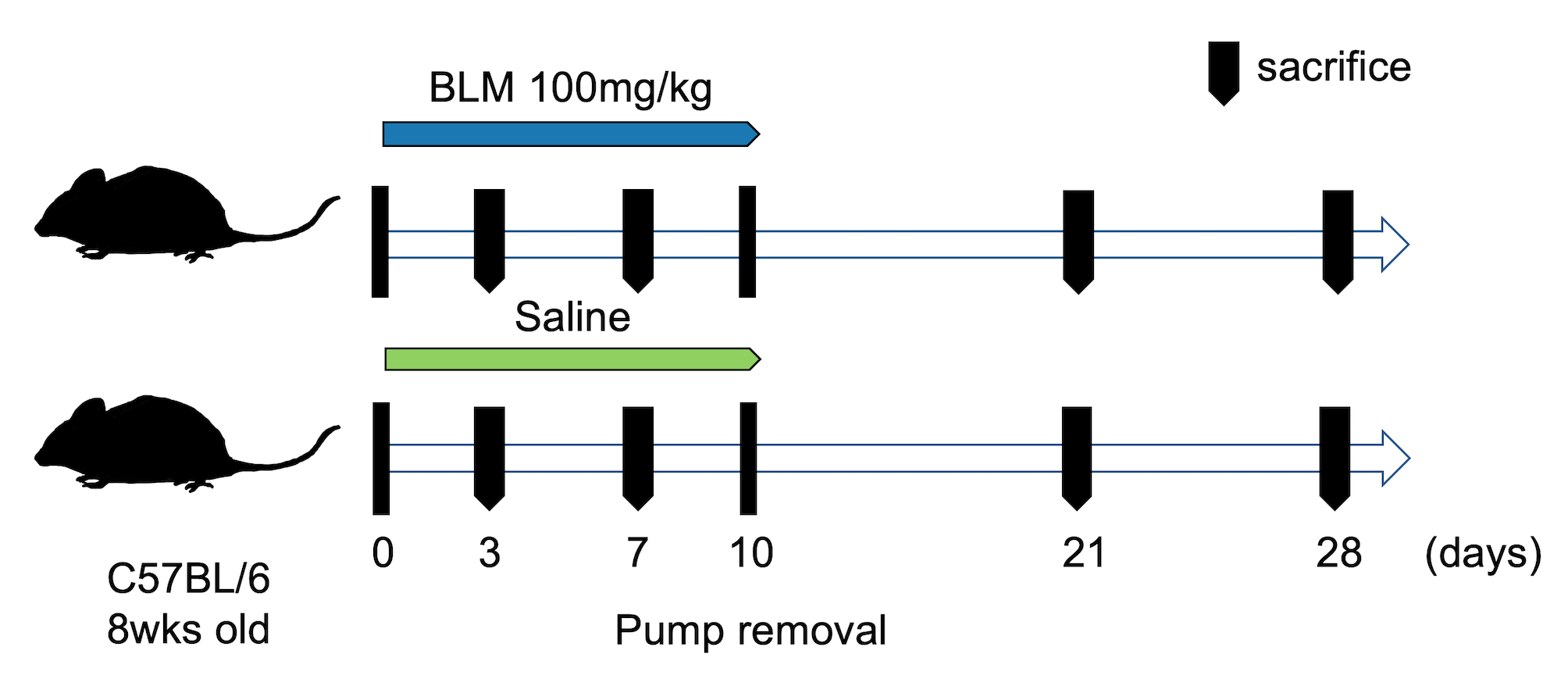

Supplement: S5 Fig — Eight-week-old male C57BL/6 mice were subcutaneously implanted with Alzet osmotic minipumps containing either a 200 μL saline vehicle or 100 mg/kg BLM at different doses. Pumps implanted under the back skin of mice slightly caudal to the scapulae were removed on day 10. BLM: bleomycin. (TIF) [file pone.0252594.s005.tif]
