# Supplementary material for: IL-18 binding protein can be a prognostic biomarker for idiopathic pulmonary fibrosis
Source: PLoS One. 2021 Jun 4;16(6):e0252594. doi: 10.1371/journal.pone.0252594 (PMC8177514; doi:10.1371/journal.pone.0252594)
Supplement: S1 Table — (DOCX) [file pone.0252594.s006.docx]

**S1 Table. Characteristics of IPF patients with lung tissue sample available**

| Case Number | 1 | 2 | 3 | 4 | 5 | 6 | 7 | 8 | 9 |
| --- | --- | --- | --- | --- | --- | --- | --- | --- | --- |
| Age (year) | 65 | 76 | 74 | 67 | 68 | 52 | 53 | 72 | 70 |
| Sex | Male | Male | Male | Male | Female | Male | Female | Male | Male |
| Smoking history,  Pack-years | 22.5 | 17.5 | 80 | 33.8 | 0 | 45 | 0 | 27.5 | 40 |
| Site of biopsies | Right | Right | Right | Right | Right | Right | Right | Right | Right |
|  | Lower | Lower | Lower | Lower | Lower | Lower | Lower | Lower | Lower |
| Days from the first consultation to SLB | 262 | 63 | 144 | 67 | 33 | 955 | 102 | 77 | 22 |
| Pathological diagnosis | UIP | UIP | UIP | UIP | UIP | UIP | UIP | UIP | UIP |
| Serum IL-18BP (ng/mL) | 5.64 | 4.82 | 4.76 | 6.06 | 5.41 | 4.77 | 4.85 | 4.58 | 3.85 |

IPF: idiopathic pulmonary fibrosis, SLB: surgical lung biopsy, IL-18BP: interleukin-18 binding protein, UIP: usual interstitial pneumonia.
